# Normalization of Blood Viscosity According to the Hematocrit and the Shear Rate

**DOI:** 10.3390/mi13030357

**Published:** 2022-02-24

**Authors:** Claudia Trejo-Soto, Aurora Hernández-Machado

**Affiliations:** 1 Instituto de Física, Pontificia Universidad Católica de Valparaiso, Casilla 4059, Chile; 2 Departament de Física de la Materia Condensada, Universitat de Barcelona, Av. Diagonal 645, 08028 Barcelona, Spain; a.hernandezmachado@gmail.com; 3 Centre de Recerca Matemàtica, Edifici C, Campus de Bellaterra, 08193 Bellaterra, Spain; 4 Institute of Nanoscience and Nanotechnology (IN2UB), Universitat de Barcelona, Av. Diagonal 645, 08028 Barcelona, Spain

**Keywords:** blood, viscosity, microfluidics, hematocrit, shear rate

## Abstract

The rheological properties of blood depend highly on the properties of its red blood cells: concentration, membrane elasticity, and aggregation. These properties affect the viscosity of blood as well as its shear thinning behavior. Using an experimental analysis of the interface advancement of blood in a microchannel, we determine the viscosity of different samples of blood. In this work, we present two methods that successfully normalize the viscosity of blood for a single and for different donors, first according to the concentration of erythrocytes and second according to the shear rate. The proposed methodology is able to predict the health conditions of the blood samples by introducing a non-dimensional coefficient that accounts for the response to shear rate of the different donors blood samples. By means of these normalization methods, we were able to determine the differences between the red blood cells of the samples and define a range where healthy blood samples can be described by a single behavior.

## 1. Introduction

The rheological properties of blood have been studied for many years, and it has been clearly demonstrated that blood has a non-Newtonian behavior [1,2,3,4,5,6,7]. This characteristic of blood is known as shear thinning, which is the property of some complex fluids to decrease their viscosity as the shear rate increases (e.g., increasing its flow velocity), and it has been widely observed in blood [3,4,8,9].

The viscosity of blood depends highly on its red blood cell (RBCs) concentration [10,11] and biomechanical properties [12,13,14], such as aggregation [6,15,16] and membrane elasticity [17,18,19]. The rise of microfluidics in the past decades has opened alternative methods to measure the rheological properties of fluids, including blood [20,21,22,23,24,25,26,27]. Several experimental and numerical studies [28,29,30,31] have analyzed the behavior of RBCs and their relation with the viscosity of whole blood using microfluidics. Moreover, some works have been published on the possibility of coupling microscopy and microfluidics for diagnostic applications [32,33,34,35,36,37]. However, the relation with blood pathologies is in many aspects an open problem for the development of reliable applications of microfluidics to Point of Care Diagnostics [38,39,40], where new approaches to rheometry using microfluidics are fundamental to create and improve PoC devices.

From a macro-rheological point of view, it is known that, the viscosity of blood is directly proportional to the hematocrit (concentration of red blood cells) [11,41], meaning that, an increase or decrease of the RBC concentration affects blood viscosity values, as well as its non-Newtonian behavior, which is lost at low hematocrit [42]. Meanwhile, from a micro-rheological point of view, blood flow is very sensitive to the elastic properties of individual RBC membranes [9,12] and to red blood cells aggregation [16,17]. These biomechanical properties have a fundamental contribution to blood viscosity. Furthermore, its has been mentioned that these properties are indicators of specific diseases related to RBCs and blood flow [43,44,45,46,47,48].

Diseases such as malaria [49,50,51], sickle cell anemia [52,53], diabetes [54], and hemolytic syndromes [55,56,57], which affect the biomechanical properties, have shown differences in the expected values of their blood viscosities as they flow at a fixed shear rate. These altered values of the viscosity are due to a decrease in the concentration of red blood cells, to an increase of the rigidity of the membrane of red blood cell, or to alterations in the aggregation process.

For this work, the viscosity of blood has been measured using a front microrheology technique [15,58]. This method consist of tracking the velocity of the blood front (i.e., blood–air interface) moving inside a microfluidic channel using a pressure-driven flow. Through a mathematical model, we relate the pressure applied to the fluid and its front velocity in the microchannel to determine the viscosity of blood.

In this article, we present a method that allows one to distinguish between healthy blood samples according to the hematocrit and shear rate. We first define an effective viscosity, $\eta_{eff}$, as a function of the blood plasma of the sample. Then, we study red blood cell crowding, presenting a normalization according to the hematocrit. In order to validate the method, we compared this normalization process with typical constitutive models from Krieger and Dougherty [59] and Quemada [60] with a non-linear dependence on the hematocrit. Later, we present a method that allows us to normalize the viscosity according to the shear rate. Finally, comparing different donor samples, with different features of their red blood cells, we obtained a range of viscosity values that can be adjusted to a single curve, using a power law model. This is the first step to compare healthy blood samples with diseased blood samples. The method presented in this work has the advantage of determining the behavior of the viscosity of blood and the effect of the hematocrit without using a specific rheological model. The method is simple and can be automatized for further diagnostics devices to assess the behavior of blood viscosity according to the response of the red blood cells to shear flow, improving diagnostic time and decreasing sample volume.

## 2. Materials and Methods

We performed experiments for healthy blood samples at 48%, 38%, and 25% hematocrit extracted from different donors, inside a rectangular microchannel, of height b = 350 μm, width w = 1 mm, and length l = 4 cm. The microchannels were fabricated in PDMS over a glass substrate. No special treatment was performed to the microchannel walls; however, after every measure, a cleaning protocol with DI water, H_2_O_2_, ethanol, and air was applied to minimize the adhesion of plasma proteins to the microchannel walls.

**Figure 1:**
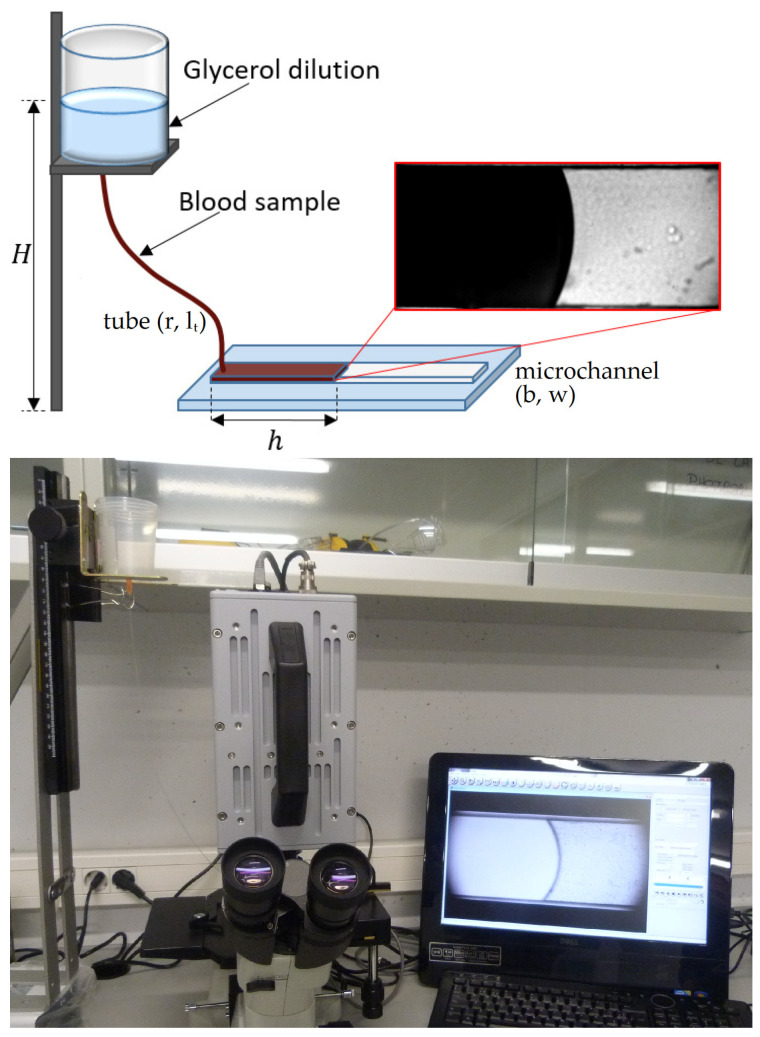
Experimental setup. On top, we present a schematic representation of the experimental set up, with a view of the blood–air interface inside the microchannel. The bottom picture shows a photograph of the experimental setup, where we can observe the reservoir, the high-speed camera, and the inverted microscope.

The observation of the blood flow inside the microchannel was made using an inverted microscope (Optika XDS-3) with a 4× objective and a high-speed camera (Photron Fastcam SA3) recording at 60 fps and 125 fps. We measured the velocity of the blood–air interface, $\dot{h}\left(t\right)$, tracking the mean front position, h(t), as a function of time between several contiguous images, Δt = 0.016 s and Δt = 0.008 s. We performed the velocity measurements at the beginning of the microchannel at a position h = 3.00 ± 0.06 mm at different injected pressures, ranging from P = 4116 ± 49 Pa to 514 ± 15 Pa. The effect of gravity inside the microchannel can be neglected, and the accumulation of particles near the meniscus did not affect the viscosity results obtained. The pressure was controlled through a fluid column inside a reservoir set at heights from H = 0.050 to 0.400 m and connected to a bio-compatible tube of uniform internal cross-section of radius r = 0.127 mm and length $l_{t}$ = 0.43 m; see Figure 1. The microfluidic devices used in these experiments were not intended to replicate the anatomical conditions of the human capillaries; therefore, the flexible and deformable characteristic of real blood capillaries was not considered in the fabrication process. A full description of the microfluidic device and details of the experimental method are reported in previous work [58].

Blood samples were extracted from anonymous healthy donors and delivered for our experiments from the *Banc de Sang i Teixit* of Barcelona, in tubes of 10 or 5 mL on an heparin based anticoagulant. We considered 3 different donors. We used a single donor to determine the hematocrit normalization and the remaining donors to compare the whole blood normalization between samples. For each donor, we used one blood sample, and we performed 5 velocity measurements at each injected pressure. The use of these samples was authorized by the Bioethics Committee of the University of Barcelona.

In order to preserve the state of the samples, these were stored on a refrigerator at 4 °C. In general, a blood sample was used within 24 h from its extraction to avoid changes in its composition, such as the emergence of echynocytes (red blood cells with an altered morphology) or hemolysis (rupture of cells), which may decrease the effective value of the hematocrit. To separate the sample in different RBCs concentrations, the blood sample in the tube was set on a centrifuge and spun for 5 min at 2500 rpm. Once the spinning was finished, the cellular fraction (RBCs, WBCs, and platelets) was confined at the bottom of the tube and the plasma on top. To avoid contamination, the samples were set on a bio-safety cabinet to equilibrate to room temperature, between 20 and 25 °C. Then, the plasma was extracted using a pipette and separated equitably in different sterilized eppendorf tubes of 2 mL, at which we added the desired erythrocyte concentration. Finally, the sample was carefully mixed using a pipette to obtain an homogeneous mixture.

By means of a 1 mL syringe, a small amount of the homogeneous mixture, V ≈ 22 μL, was introduced inside the tube that communicates the fluid recipient with the microchannel, until it was completely filled with the sample. To avoid the effects of RBC sedimentation inside the recipient, the container was filled with glycerol diluted in distilled water at a concentration of 20%. The resultant dilution had a viscosity of 1.93 mPas and an approximate density of the blood sample, ρ = 1050 kg/m^3^. Since glycerol at 20% concentration is less viscous than blood, we performed the measures at the beginning of the microchannel, where the pressure drop due to glycerol is negligible, and we ensure that the fluids do not mix [58]. It is important to note that the blood samples were carefully mixed to disaggregate cells before introducing it into the tube to disperse all pre-existing aggregates. The time scale of the experiment was small enough (≈20 s) to avoid sedimentation playing a significant role inside the microchannel. All of the samples were extracted the day of the experiments.

## 3. Theoretical Model

According to a power law model for fluids, the non-Newtonian character of a fluid is described using the following equation [61],

(1) $$\eta \left(\dot{\gamma }\left(z\right)\right)=m\dot{\gamma }{\left(z\right)}^{n-1},$$

where *n* is a constant that depends on the fluid, *m* is a prefactor (known as consistency index) that may depend on *n* [15], $\dot{\gamma }\left(z\right)=\frac{\partial v_{x}\left(z\right)}{\partial z}$ is the shear rate, and $v_{x}\left(z\right)$ is the velocity of the fluid as a function of the vertical position *z* in the microchannel. Averaging the microchannel depth *b* to obtain the mean shear rate of the front, we defined the shear rate as $\dot{\gamma }=\dot{h}/b$, where $\dot{h}$ is the averaged velocity of the front. Therefore, the viscosity as a function of the velocity averaged along the microchannel depth can be written as

(2) $$\eta =m{\dot{\gamma }}^{n-1}.$$

Considering the coupled system reservoir–tube–microchannel, the pressure difference inside the microchannel is

(3) $$\Delta P=\rho gH-\Delta P_{t}-P_{cap},$$

where ρgH is the hydrostatic pressure, $\Delta P_{t}$ is the pressure drop inside the tube, and $P_{cap}$ is the capillary pressure inside the microchannel.

The capillary pressure is calculated by means of the Young–Laplace equation for a rectangular channel

(4) $$P_{cap}=2\tau \cos\theta \left(\frac{1}{b}+\frac{1}{w}\right),$$

where τ is the surface tension of the blood–air interface as a function of the hematocrit and temperature [62], and θ is the contact angle between the fluid and the microchannel walls, which is calculated for each sample at every injected pressure. The capillary pressure values range from 100 Pa to 300 Pa.

The flow rate inside the microchannel can be defined as

(5) $$\begin{array}{ccc} Q & = & w\int_{-b/2}^{b/2}v_{x}\left(z\right)dz,\\ Q & = & 2w{\left(\frac{\Delta P}{mh(t)}\right)}^{\frac{1}{n}}{\left(\frac{b}{2}\right)}^{\frac{1}{n}+2}\left(\frac{1}{\frac{1}{n}+2}\right), \end{array}$$

where we have taken into account that $v_{x}\left(z\right)$ and ΔP can be obtained from

(6) $$\dot{\gamma }\left(z\right)=b{\left(\frac{\Delta P}{mh(t)}z\right)}^{\frac{1}{n}}$$

and $v_{x}\left(z=\pm b/2\right)=0$.

From Equation (5), we derived an average front velocity, $\dot{h}=\langle v_{x}\left(z\right)\rangle$ so that $Q=bw\dot{h}$. Taking advantage of the relation between Q and $\dot{h}$, we can write a relation between ΔP and $\dot{h}$ as

(7) $$\rho gH-\Delta P_{t}-P_{cap}=\frac{2m}{b}h{\left[\frac{2}{b}\left(2+\frac{1}{n}\right)\right]}^{n}{\dot{h}}^{n},$$

and considering that the pressure drop inside the tube can be written as

(8) $$\Delta P_{t}=\frac{2l_{t}m}{r^{n+1}}{\left(\frac{1}{n}+3\right)}^{n}v_{t}^{n}.$$

According to mass conservation, $v_{t}=wb\dot{h}/\pi r^{2}$, and substituting Equation (8) in Equation (7), we obtain [15]

(9) $$\rho gH-P_{cap}=m\left[\frac{2l_{t}{\left(\frac{1}{n}+3\right)}^{n}}{r^{1+n}}{\left(\frac{bw}{\pi r^{2}}\right)}^{n}+h\left(t\right)\frac{{\left(2+\frac{1}{n}\right)}^{n}}{{\left(\frac{b}{2}\right)}^{1+n}}\right]{\dot{h}}^{n}\left(t\right).$$

Under the conditions of our experimental setup, the resistance of the tube is much larger than the resistance of the microchannel, and the second term of Equation (9) is negligible. Then, $\dot{h}$ is constant, and Equation (9) reduces to

(10) $$\rho gH-P_{cap}=m\frac{2l_{t}{\left(\frac{1}{n}+3\right)}^{n}}{r^{1+n}}{\left(\frac{bw}{\pi r^{2}}\right)}^{n}{\dot{h}}^{n}.$$

We define a parameter K(m,n) as a generalized consistency index, where

(11) $$K\left(m,n\right)=m\frac{2l_{t}{\left(\frac{1}{n}+3\right)}^{n}}{r^{1+n}}{\left(\frac{b^{2}w}{\pi r^{2}}\right)}^{n}.$$

We adapted the consistency index from the power law model to adjust it to our experimental configuration and to obtain the viscosity of the fluid directly from our experimental results.

Combining Equations (10) and (11) and defining an effective pressure $\Delta P_{eff}=\rho gH-P_{cap}$, we can describe a non-linear relation between the effective pressure imposed to the fluid and the associated velocity of the fluid front, as

(12) $$\Delta P_{eff}=K\left(m,n\right){\left(\frac{\dot{h}}{b}\right)}^{n},$$

where the fluid is shear thinning when n<1, shear thickening when n>1, and Newtonian if n=1. The parameters *K* and *n* are obtained from the experimental results. To determine the value of the prefactor *m*, we use

(13) $$m=\frac{K}{\frac{2l_{t}{\left(\frac{1}{n}+3\right)}^{n}}{r^{1+n}}{\left(\frac{b^{2}w}{\pi r^{2}}\right)}^{n}},$$

where *m* is determined as a function of *K* and *n*.

A comparison of our theoretical approach with the Weissenberg–Rabinowitsch–Mooney correction for slit rheometry is presented in Appendix A.

## 4. Results

### 4.1. Effective Viscosity

**Figure 2:**
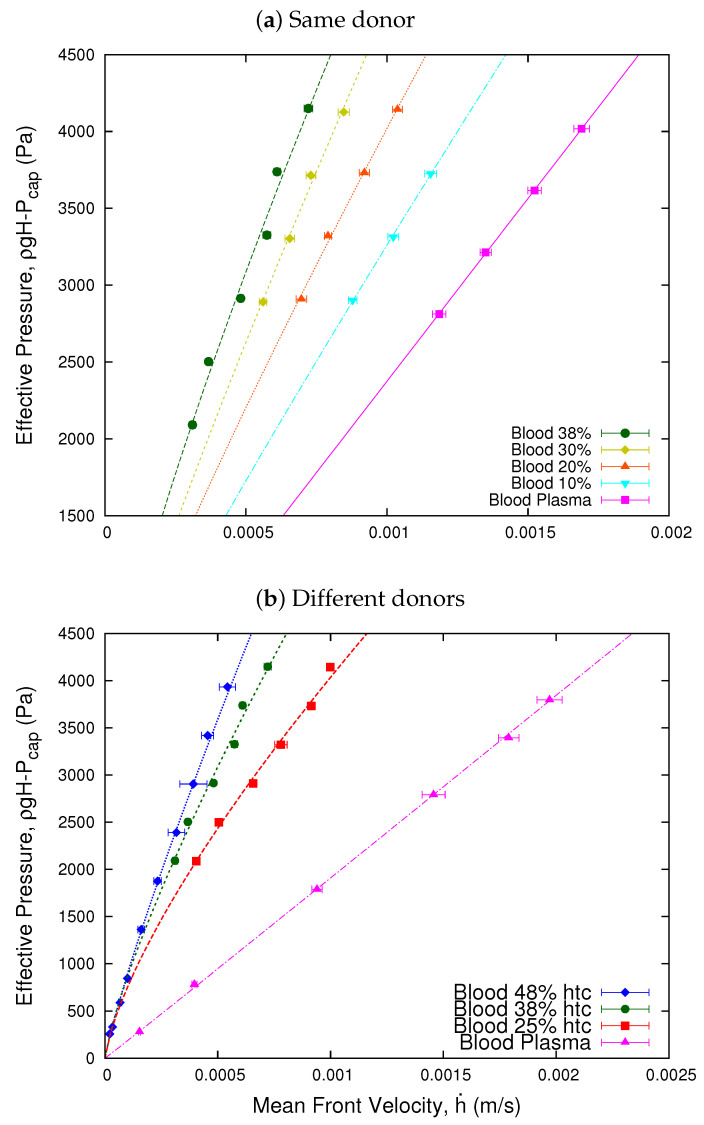
Effective pressure vs. mean front velocity. The figure shows the effective pressure $\Delta P_{eff}=\rho gH-P_{cap}$ as a function of the velocity of the blood front. Plot (**a**) Same donor shows the viscosity of a single 38% hematocrit blood sample separated in different concentrations of erythrocytes. For the plasma sample, the value of the exponent is n = 1.001, indicating its Newtonian behavior. The values of the exponents of the samples vary from n = 0.80 to n = 0.91 for the 10% sample, which indicates that the samples decrease their shear thinning behavior for low hematocrit. Plot (**b**) Different donors shows the results for samples extracted from different donors, according to a power law fit. For the plasma sample of the 48% hematocrit, the value of the exponent n = 1.01 indicates its Newtonian behavior. Meanwhile, for the blood samples, the exponents range from n = 0.86 to n = 0.73, indicating that all the samples present a shear thinning behavior. The values of the exponents *n* for each curve were obtained fitting Equation (12) to the experimental results.

We represent the pressure difference applied to the fluid as an effective pressure $\Delta P_{eff}=\rho gH-P_{cap}$, which is defined as a function of the hydrostatic pressure, ρgH, and the capillary pressure, $P_{cap}$, due to the curvature of the blood interface. Figure 2 shows the relation between the effective pressure, $\Delta P_{eff}$, as a function of the mean front velocity $\dot{h}$, of the blood samples compared with a plasma sample. Here, we observe that the relation presents a non-linear character which, according to the power law model for fluids, defines the shear thinning behavior of blood and the Newtonian character of blood plasma. The value of the exponent *n* is obtained fitting Equation (12) to our experimental results.

Once we have established the shear thinning behavior of blood, we defined an effective viscosity value for the blood samples, as the relation between the pressure exerted on the fluids and its velocity response [35]. We define the value of the viscosity of blood relative to its plasma as an effective viscosity,

(14) $$\eta_{eff}=\frac{{\dot{h}}_{p}\Delta P_{eff}}{\dot{h}\Delta P_{eff_{p}}},$$

where $\Delta P_{eff}$ and $\Delta P_{eff_{p}}$ are the effective pressures for the blood sample and its blood plasma, respectively, and $\dot{h}$ and ${\dot{h}}_{p}$ are the mean front velocities of the blood samples and its blood–plasma interface. The geometrical parameter of the experimental setup and microchannel remained constant during the experiments. This effective viscosity does not reflect the actual value of the viscosity of the sample but rather the value of the viscosity normalized to its own plasma viscosity for different velocity responses of the sample. This definition has the advantage to determine a point by point viscosity without any mathematical model.

### 4.2. Normalization of Blood Viscosity According to the Hematocrit

#### 4.2.1. Effective Viscosity and Red Blood Cells Concentration of the Same Donor: A Linear Approach

**Figure 3:**
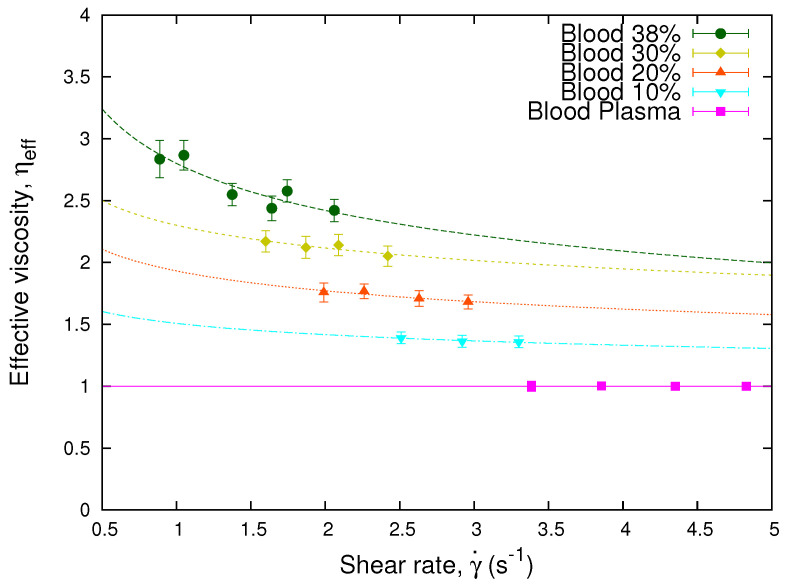
Effective viscosity vs. shear rate. The plot shows the effective viscosity, obtained through Equation (14), as a function of the shear rate, $\dot{\gamma }=\dot{h}/b$, of a blood sample separated in different RBCs concentrations and blood plasma. The lines represent the fits obtained through Equation (2) for the different effective viscosities. The values of the exponents of the samples are n = 0.80 for the 38% sample, n = 0.87 for the 30% sample, n = 0.87 for the 20% sample, and n = 0.91 for the 10% sample, which coincide with those obtained in Figure 2.

It is widely known that the non-Newtonian behavior of blood viscosity depends on several properties such as concentration, aggregation, and membrane elasticity of its RBCs. In this subsection, we will focus on the concentration (hematocrit), considering the sample of a single donor. We study the effective viscosity of the sample as a function of the shear rate for different values of the hematocrit. We divided a blood sample of 38% hematocrit in different red blood cell concentration of 30%, 20%, and 10%. Then, using Equation (14), we determined the effective viscosity of the original sample and its decreased hematocrit preparations. Figure 3 shows the measured effective viscosities of the 38% hematocrit blood sample and its different red blood cells concentrations. We observe that the effective viscosity values decrease according to the decrease of the red blood cells concentration. The curves represent the fits obtained through a power law, which is analogous to Equation (2), for the different effective viscosities. Here, we observe that the power law exponent *n* decreases while the hematocrit increases, and its value coincides with those obtained in Figure 2.

**Figure 4:**
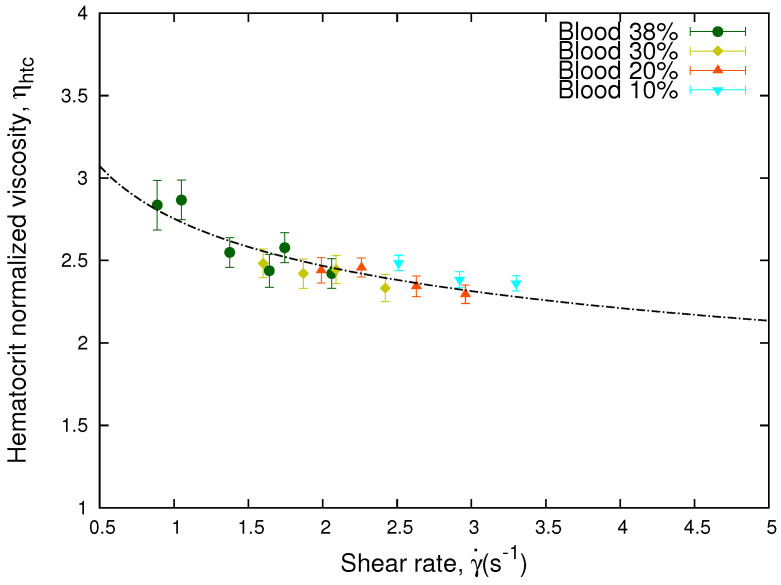
Hematocrit normalized viscosity vs. shear rate. The figure shows the hematocrit normalized viscosity, $\eta_{htc}$, of the same blood sample as a function of the shear rate. The curves were collapsed onto a single curve according to Equation (15) using $\phi_{control}$ = 0.38. The line represent the fit obtained through Equation (2) for the hematocrit normalized viscosity. The value of the parameters are m = 2.75 and n = 0.84.

The curves of the viscosity for different hematocrits as a function of the shear rate show the sensitivity of the viscosity to hematocrit. As shown in Figure 4, these curves collapse when the relative effect of hematocrit differences are taken into account by means of the following equation,

(15) $$\eta_{htc}=1+\left(\eta_{eff}-1\right)\frac{\phi_{control}}{\phi }.$$

Here, ϕ are the different hematocrits of the sample and $\phi_{control}$ is an arbitrary value and only implies a vertical displacement in the values of the curve of hematocrit normalized viscosity $\eta_{htc}$ as a function of the shear rate. It is inferred from Equation (15) that when the control hematocrit, $\phi_{control}$, and the sample hematocrit, ϕ, are equal, the hematocrit normalized viscosity corresponds to the effective viscosity, $\eta_{htc}=\eta_{eff}$.

In Figure 2a, we observe that for a fixed value of the effective pressure, we obtain smaller values of the shear rates as the hematocrit increases. In general, the effective viscosity is a non-linear function of the erythrocytes concentration (ϕ) and the shear rate ($\dot{\gamma })$,

(16) $$\eta_{eff}\left(\phi ,\dot{\gamma }\right)=1+f\left(\phi ,\dot{\gamma }\left(\phi ,\Delta P_{eff}\right)\right).$$

According to our experimental results, for low hematocrit (≤30%) and high shear rates, we obtain from Figure 3 that the function *f* does not depends on the shear rate, giving an approximate Newtonian behavior, and *f* scales linearly with the hematocrit. Therefore, $\eta_{htc}$, defined by Equation (15), does not depend on the erythrocytes concentration, and the curves collapse onto a master curve. Figure 4 shows how using Equation (15), the different values of the viscosity at different hematocrits collapse onto a single curve. If this collapse occurs, it means that the different values of the effective viscosity of the samples are solely due to the hematocrit.

The expression in Equation (15) was originally used to extrapolate the apparent viscosity of blood to the viscosity of a 45% hematocrit sample inside small capillaries [63]. We have adapted the original expression from Ref. [63] to define a hematocrit normalized viscosity of blood according to the concentration of RBCs, which allows us to obtain a single collapsed curve. We obtained that for our front microrheology experiments, at the pressure and shear rate ranges studied, Equation (15) gives a master curve for all the hematocrits considered.

**Figure 5:**
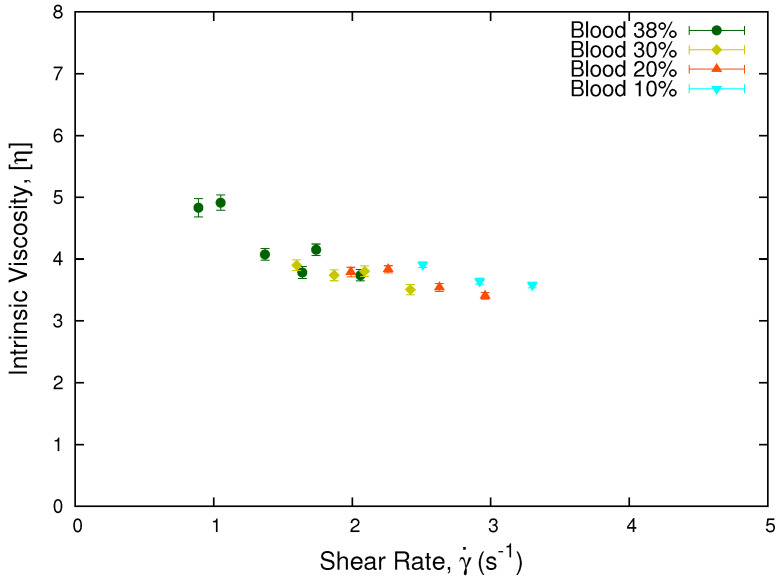
Intrinsic viscosity vs. shear rate. The plot show the collapse of the intrinsic viscosity values for a sample from a single donor separated in different hematocrits. The normalization was performed using the linear approximation of the Krieger and Dougherty model, Equation (17), being consistent with the collapse observed in Figure 4.

Other models have been developed to study the non-linear scaling of a suspension of particles, such as the Krieger and Dougherty model [59] and the Quemada model [60]. The linear approximation for these models is expressed as the following equation,

(17) $$\eta_{eff}=1+\left[\eta \right]\phi ,$$

where $\eta_{eff}$ is obtained through Equation (14), and ϕ is the erythrocytes concentration of each sample. Plotting the intrinsic viscosity $\left[\eta \right]$, see Figure 5, we obtain that the viscosity values for different hematocrits from the same donor collapse onto a single curve. This result is in agreement with our results using Equation (15) for a single donor and low hematocrits.

Is important to note that the non-linearity of Equation (15) is implicit in the effective viscosity as a function of the hematocrit and the shear rate, as obtained through Equation (14). According to Equation (16), if the function $f(\phi ,\dot{\gamma )}$ is non-linear, (15) will be non-linear as well. For the Newtonian case from Equations (15) and (17), the intrinsic viscosity is

(18) $$\left[\eta \right]=\frac{\eta_{htc}-1}{\phi_{control}}$$

and one obtains the master curves from Figure 4 and Figure 5.

#### 4.2.2. Viscosity and Red Blood Cell Concentration for Different Donors

**Figure 6:**
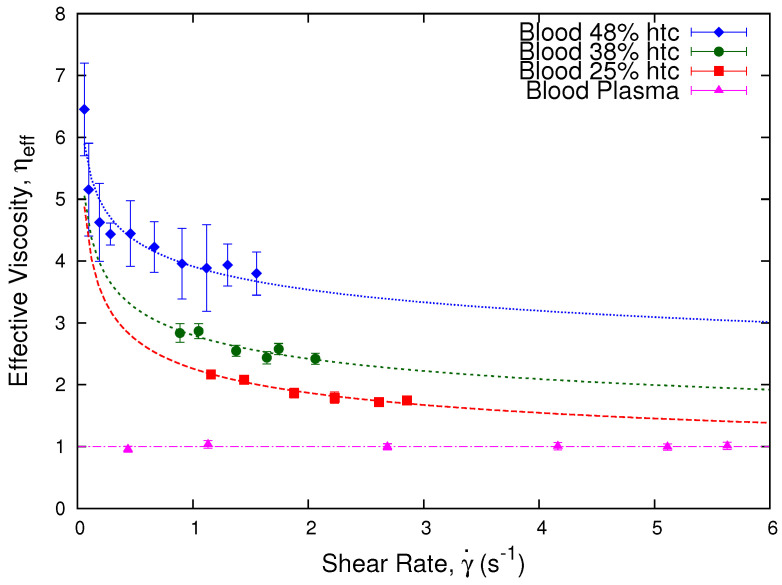
Effective viscosity vs. shear rate. The plot shows the effective viscosity, $\eta_{eff}$, which was obtained through Equation (14) as a function of the shear rate $\dot{\gamma }$ (s^−1^). We present three different blood samples at 48%, 38%, and 25% hematocrit, from different donors: *A*, *B*, and *C*, respectively. The curves show the shear thinning tendencies of the three samples and the Newtonian condition of the blood plasma of the 48% hematocrit sample. As well, we observe how the effective viscosity values are affected by the hematocrit of the samples. The dashed lines are a guide to the eye, which are obtained through Equation (2).

In Figure 6, we show how the effective viscosity of blood varies as a function of the shear rate inside the microchannel for three different donors at erythrocyte concentrations of 48%, 38%, and 25%. The effective viscosity is obtained through Equation (14) relative to the plasma of each sample. We observe that each sample presents shear thinning behavior while blood plasma is Newtonian. As well, we observe that each sample presents different viscosity values.

**Figure 7:**
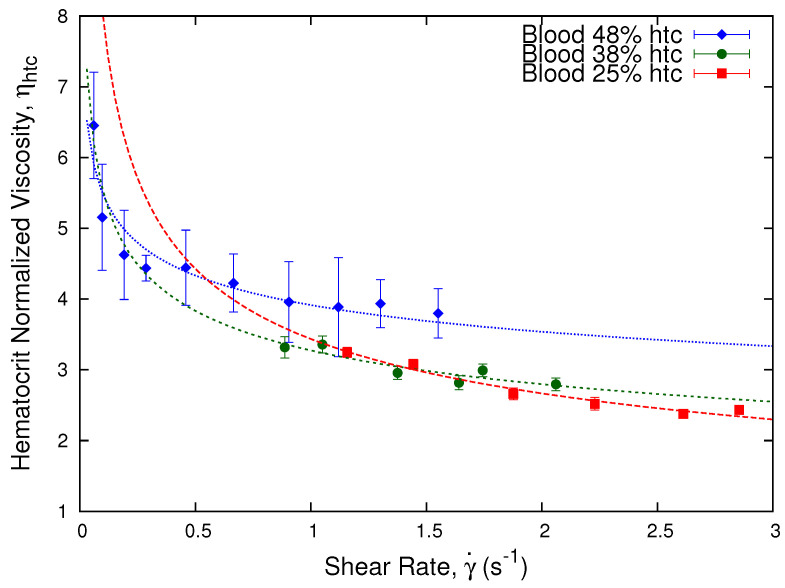
Hematocrit normalized viscosity vs. shear rate. Plot showing the hematocrit normalized viscosity, $\eta_{htc}$, as a function of shear rate for blood samples from different donors: donor *A* with a 48% hematocrit, donor *B* with a 38% hematocrit, and donor *C* with a 25% hematocrit, according to Equation (15) as a function of $\phi_{control}$ = 0.38.

Following the procedure of the previous section, we obtained the hematocrit normalized viscosity from the effective viscosity of the blood samples for different donors. This normalization procedure allows us to compare different samples even if their original hematocrit is different, minimizing the intervention on the samples. Moreover, it allows us to focus on the different properties of the red blood cells instead of only their concentration, since we are able to distinguish the difference between the viscosity of blood samples disregarding the effect of the hematocrit, and highlighting the differences between the cells from different donors. In Figure 7, we show the obtained hematocrit normalized viscosity, $\eta_{htc}$, as a function of the shear rate using Equation (15).

We observe that the hematocrit normalization, as shown in Equation (15), shows differences in the viscosity values of the samples depending on their red blood cells concentration. The process is enough to collapse the viscosity curves of the 38% and 25% hematocrit, while the 48% is slightly off.

**Figure 8:**
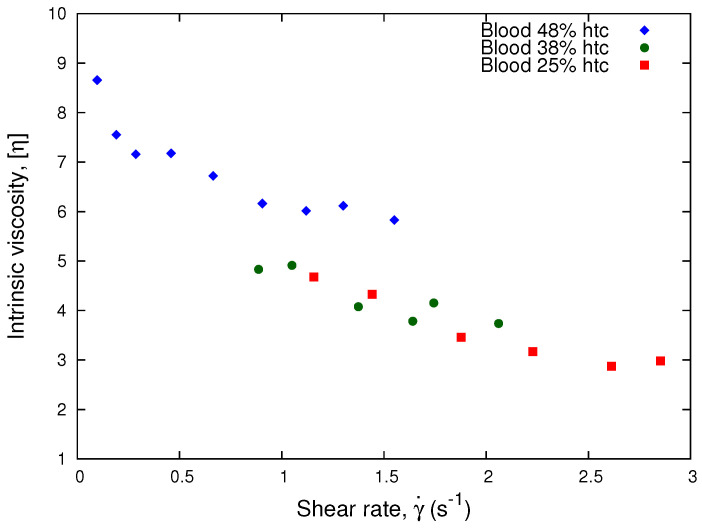
Intrinsic viscosity vs. Shear Rate. The plot shows the normalization process performed using the linear approximation of the Krieger and Dougherty model, Equation (17), for different donor samples.

According to Equation (17), we plot the intrinsic viscosity, $\left[\eta \right]$, as a function of the shear rate. In Figure 8, we observe that the curves of the 38% and 25% hematocrits collapse. This implies that our results for low hematocrits are approximately Newtonian. Therefore, the Krieger and Dougherty model is consistent with Equation (15) for low hematocrit and high shear rates, which is in agreement with our results from Figure 7.

Since the effective viscosity values obtained in Figure 6 are obtained from Equation (14), they present a non-linear behavior; therefore, according to Equation (16), the function $f(\phi ,\dot{\gamma })$ is non-linear. We will compared this approach with other non-linear models in the following section.

#### 4.2.3. Non-Linear Scaling According to the Hematocrit

We have analyzed a generalization of Equation (17) for higher hematocrits, based on the typical constitutive models from Krieger and Dougherty [59] and Quemada [60] with a non-linear dependence on the hematocrit. The Krieger and Dougherty model states that

(19) $$\eta_{eff}=\frac{\eta }{\eta_{p}}={\left[1-\frac{\phi }{\phi_{max}}\right]}^{-\left[\eta \right]\phi_{max}},$$

where $\eta /\eta_{p}$ is known as the relative viscosity. This relative viscosity is equivalent to the effective viscosity obtained through Equation (14), which is a function of the concentration of RBCs of the sample ϕ, the maximum packing fraction $\phi_{max}$, and an intrinsic viscosity $\left[\eta \right]$.

For deformable red blood cells, $\phi_{max}\approx 1$[64,65]. In the Newtonian case, the intrinsic viscosity does not depend on the shear. According to Quemada (1978) [60], introducing $\left[\eta \right]=2/\phi_{max}$ in Equation (19), the Krieger and Dougherty model reduces to the Quemada model, as shown in Equation (20).

This last model proposes that the non-Newtonian properties observed in steady-state shear flow experiments can be described with the help of an effective viscosity, $\eta /\eta_{p}$. This viscosity is a function of the hematocrit, ϕ, of the sample and an effective intrinsic viscosity $\left[\eta \right]$, which depends on the shear rate $\dot{\gamma }$ [66],

(20) $$\eta_{eff}=\frac{\eta }{\eta_{p}}={\left[1-\frac{\phi \left[\eta \right]}{2}\right]}^{-2}.$$

For the non-Newtonian case, Equation (20), the intrinsic viscosity depends on the shear rate through the effective viscosity, Equation (14). Solving Equation (20) for the intrinsic viscosity, [η], we obtain

(21) $$\left[\eta \right]=\frac{2}{\phi }\left[1-{\left(\frac{1}{\eta_{eff}}\right)}^{1/2}\right].$$

**Figure 9:**
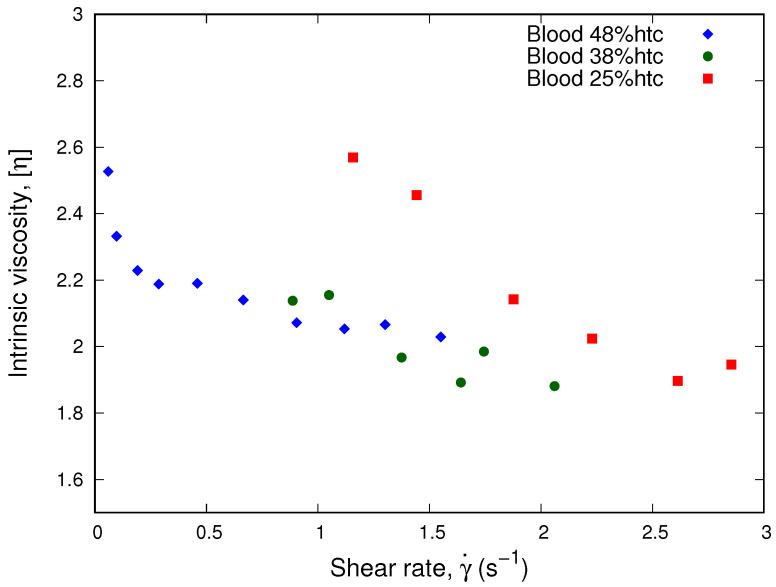
Intrinsic viscosity vs. shear rate. The plot shows the intrinsic viscosity obtained from the Quemada model, as shown in Equation (20). We observe how the value of the intrinsic viscosity approaches 2 at high shear rates corresponding to the maximum packing fraction when blood viscosity is close to Newtonian.

Plotting this intrinsic viscosity as a function of the shear rate in Figure 9, the dependence on the shear rate is implicit in the values of the effective viscosity. We observe how the values of the intrinsic viscosity for all samples approaches 2 at high shear rates corresponding to the maximum packing fraction when blood viscosity is close to Newtonian. We observe that a collapse occurs at low shear rates and high hematocrit for the 48% and the 38% sample; however, at low shear rates, the 25% sample does not fit in this new collapse.

Even though the models collapse the curves separately for low hematocrit and high shear rates, as shown in Figure 7 and Figure 8, and for high hematocrit using the Quemada model, as shown in Figure 9, we propose a new parameter to determine a universal master curve that accommodates the full spectrum of the three healthy blood samples, low hematocrit, and high shear rates samples, as well as high hematocrit and low shear rates.

### 4.3. Normalization of Blood Viscosity for Different Donors According to the Shear Rate

In this section, we propose a general framework for low shear rates and high hematocrit where the non-linear behavior of the viscosity is associated to the shear rate response of different donors samples. We define a characteristic non-dimensional number, which expresses the ratio between a characteristic relaxation time, $\tau_{m}$, associated to the biomechanical properties of the suspended cells in the fluid and $\tau_{\eta }$, which is a characteristic viscous time associated with the viscous forces of the fluid [67,68]

(22) $$C_{0}=\frac{\tau_{m}}{\tau_{\eta }},$$

where $\tau_{m}={\left(\kappa {\dot{\gamma }}_{0}\right)}^{-1}$ and $\tau_{\eta }={\dot{\gamma }}^{-1}$; then, the characteristic number, $C_{0}$, is defined as the ratio between the shear rate $\dot{\gamma }=\dot{h}/b$ s^−1^ of the sample and an effective shear rate ${\dot{\gamma }}_{0}$ as

(23) $$C_{0}=\frac{\dot{\gamma }}{\kappa {\dot{\gamma }}_{0}},$$

where ${\dot{\gamma }}_{0}=E_{0}/\eta_{p}d^{3}$. $E_{0}$ is the bending energy of a healthy RBC membrane ($E_{0}\approx 50k_{B}T=2\times 10^{-19}$ J), $\eta_{p}$ is the viscosity of the plasma of each sample, d ≈ 7.8 μm is the average diameter of the red blood cell, and κ is a dimensionless factor that accounts for the relative variations of the effective shear rate according to the different donors.

The value for $E_{0}$ has been estimated through different methods such as micropipette [69], AFM [70], and optical tweezers [71]. The parameter $E_{0}$ accounts for the biomechanical properties of a healthy unaggregated RBC. If the blood samples contains healthy RBCs with the same bending rigidity and no aggregation, then κ=1.

Estimating the shear rate ratios between the different blood samples as a function of a control hematocrit blood sample, we intend to collapse their viscosity curves as a function of the parameter $C_{0}$, in order to determine a master curve for all three blood samples. The more similar to 1 is κ, the closer to the master curve is the viscosity of the sample.

To perform the normalization process, we start from the hypothesis that for an approximate superposed viscosity value, $\eta_{htc}$, from Figure 7 of samples *A* and *B*, we can find a characteristic number, $C_{0}$, which satisfies the condition

(24) $$C_{0_{A}}=C_{0_{B}}.$$

According to this hypothesis, a characteristic number is associated to the shear rate at which the viscosities have close values. Hence, using the definition from Equation (23), and considering that the difference in diameter between *A* and *B* red blood cells is negligible ($\delta d/d=10^{-2}$), we obtain the following relation between the shear rate ratios of the different blood samples from donors *A* and *B*, as

(25) $$\frac{\kappa_{A}}{\kappa_{B}}=\frac{\eta_{p_{A}}}{\eta_{p_{B}}}\frac{1}{N}\sum_{i=1}^{N}\frac{{\dot{\gamma }}_{A_{i}}}{{\dot{\gamma }}_{B_{i}}}.$$

Comparing the shear rate ratios from a pair of samples *A* and *B*, if the ratio $\kappa_{A}/\kappa_{B}$ from Equation (25) is different from 1, it indicates a different response of the viscosity to the shear rate from different donors. The values of $\eta_{p_{A}}$ and $\eta_{p_{B}}$ are the respective plasma viscosity of each sample, and *N* is the number of values where the viscosity difference between samples was δη < ±0.5, according to the experimental error of the measurements. Therefore, the relation between the coefficient κ between the samples will be obtained according to the relation between their averaged shear rates $\dot{\gamma }$ and the factor obtained from $\eta_{p_{A}}/\eta_{p_{B}}$.

It is important to note that since the curves for different donors do not collapse in Figure 7, the difference of viscosities obtained for different samples can be interpreted as the sensitivity of the viscosity to the shear rate due to the different origins of the samples (different donors). Since we have already normalized the viscosities according to the hematocrit of the samples from different donors, the concentration of RBCs does not affect the normalized viscosity values.

From Figure 7, we chose arbitrarily the sample of donor *B* with 38% hematocrit as the control sample ($\kappa_{B}=1$) and compared it to the viscosity values of the samples from donor *A* with a 48% hematocrit and donor *C* with a 25% hematocrit. According to Equation (25), we compared samples from donor *B* with donors *A* and *C*, $\kappa_{A}/\kappa_{B}$ = 1.4 ± 0.2 and $\kappa_{C}/\kappa_{B}$ = 0.6 ± 0.2. Therefore, the coefficients κ of donor *A* sample and donor *C* sample can be written as a function of the coefficient κ of the donor *B* sample as

(26) $$\begin{array}{c} \kappa_{A}=1.4\kappa_{B}, \end{array}$$

(27) $$\begin{array}{c} \kappa_{C}=0.6\kappa_{B}. \end{array}$$

**Figure 10:**
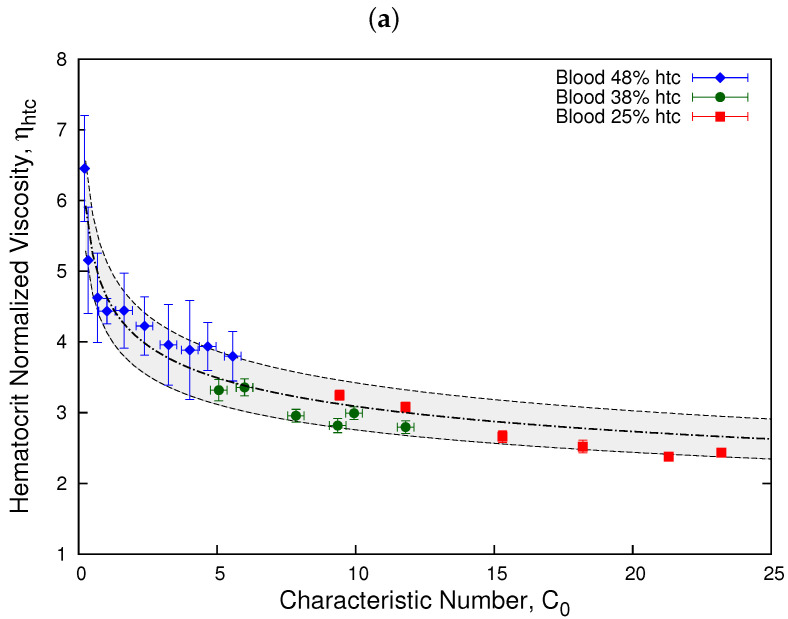

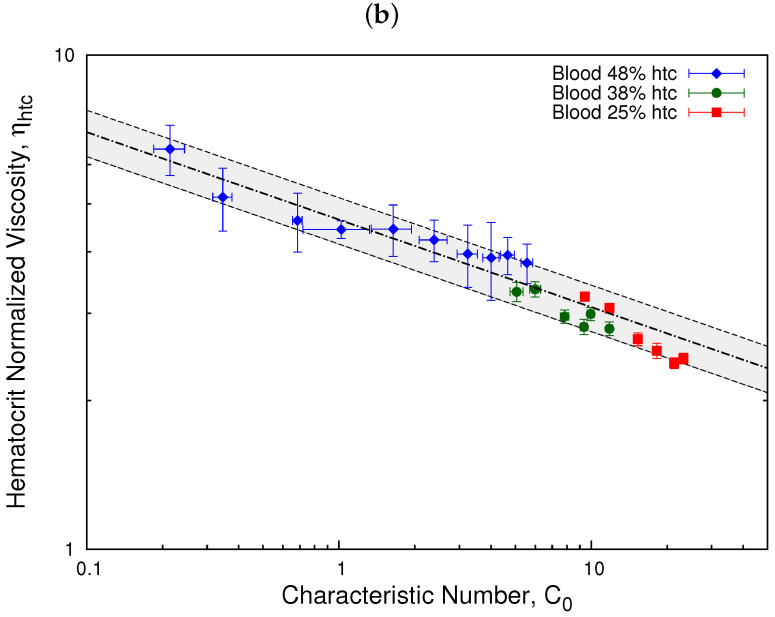
Hematocrit normalized viscosity vs. characteristic number. The plots show the hematocrit normalized viscosity as a function of the characteristic number, $C_{0}$, for donors *A* and *C* as a function of the shear rate of donor *B*. Plot (**a**) shows the results for a linear scale and plot (**b**) for a log scale. The relations are given as $\kappa_{A}=1.4\kappa_{B}$ and $\kappa_{C}=0.6\kappa_{B}$. The gray zone represents the interval where all three samples are considered part of the master curve according to its viscosity error δη = ±0.5. The dashed-dotted line represents the fit obtained through Equation (2) for the hematocrit normalized viscosity. The value of the parameters are m = 4.64 and n = 0.83.

In Figure 10, we show the results obtained for $\eta_{htc}$ for different donors by normalizing according to the coefficient κ of donor *B* control sample, $\kappa_{B}$. Here, we clearly observe that all three samples exhibit a similar behavior and can be fitted onto a master curve. This result allows us to determine a range where healthy blood samples can be described by a single behavior, which can be used to distinguish them from diseased blood samples. The gray zone represents the interval where all three samples are considered part of the master curve. The line represent the fit obtained through Equation (2) for the hematocrit normalized viscosity. The value of the parameters are m = 4.64 and n = 0.83. Moreover, Figure 10 shows how the difference of viscosities obtained for different samples in Figure 7 is related to the sensitivity of the viscosity of blood from different donors, as represented by factor κ.

According to the results from Figure 4, the value of the power law index n = 0.84 is compatible with the master curve results from Figure 10, n = 0.83. Additionally, we have determined the ratio between shear rates comparing the values of the intrinsic viscosity obtained with the Quemada model (Figure 9) for high hematocrit samples 48% (A) and 38% (B). We determined, using Equation (25), that the relation between the coefficients κ is similar to the one obtained using the normalization from Equation (15)

(28) $$\kappa_{A}=1.47\kappa_{B}.$$

## 5. Discussion

The rheological properties of blood depend highly on the properties of its red blood cells concentration and biomechanical properties, such as membrane elasticity and aggregation. In this work, we have obtained experimentally the viscosity of blood, tracking the velocity of a blood–air interface inside a microfluidic channel, using a pressure-driven flow. We have defined an effective viscosity as a function of the blood plasma of the samples, and we have been able to observe that our experimental method reproduces the Newtonian behavior of the blood plasma as well as the shear thinning behavior for blood.

Our method is able to distinguish blood samples with different hematocrits for a single donor and several donors. For a single donor, we obtained that our method successfully normalizes the viscosity of blood according to the hematocrit, where the viscosity values collapse onto a master curve.

Performing the normalization according to the hematocrit for different donors, we observe an approximate collapse between the effective viscosity values of the different samples. When the hematocrit normalized viscosity of different samples collapse, we infer that the differences in their effective viscosities are mainly due to their different RBCs concentration. If the viscosity curves do not collapse well, we inferred that the response of the viscosity to the shear rate are different.

In the case of different donors, we performed a second normalization of the viscosity according to the shear rate. We introduced a non-dimensional coefficient, which expresses the ratio between the biomechanical properties of the suspended cells in the fluid and the viscous forces of the fluid. We were able to adjust a master curve according to a characteristic number as a function of the shear rate ratios between different donors samples.

Since we used the samples just as they were extracted without any intervention on them, the analysis presented requires knowing the hematocrit of the samples before hand. However, this is not an issue, because hematocrit is a standard clinical analysis, and this information is easy to collect. Furthermore, the method allows manually intervening the experimental samples to compare different donors with the same hematocrit.

## 6. Conclusions

Combining both methods presented in this work, normalization according to the hematocrit and normalization according to the shear rate, we were able to determine differences between the red blood cells of the samples. For different donors, the collapse of the viscosity values onto a master curve indicates that the normalization procedure captures the relevant mechanism controlling blood viscosity. This result allows us to determine a range where healthy blood samples can be described by a single behavior.

These methods have the advantage of determining the behavior of the viscosity of blood and the effect of the hematocrit without using a specific rheological model and disregarding the effect of blood plasma viscosity. Furthermore, the methods are simple and can be automatized and included in the development of diagnostics devices to assess the behavior of blood viscosity according to the response of the red blood cells to shear flow, improving diagnostic time and decreasing sample volume. Moreover, these normalization processes could also be extended to differentiate between healthy and diseased blood samples. We are currently working on the automatization of the measurements and procedures to determine the normalization curves, and we are performing experiments with diseased blood samples where the biomechanical variations of the red blood cells are known.

## Data Availability

Data are contained within the article.

## Glossary

*Q*	Flow rate	$\frac{m^{3}}{s}$ or $\frac{\mu L}{\min}$
η	Viscosity	Pa·s
ρ	Density	$\frac{\mathrm{kg}}{m^{3}}$
$\dot{\gamma }$	Shear rate	s^−1^
σ	Shear stress	Pa
∇P	Pressure gradient	Pa/m
ΔP	Pressure drop	Pa
$P_{cap}$	Capillary pressure	Pa
$P_{hyd}=\rho gH$	Hydrostatic pressure	Pa
*g*	Acceleration of gravity	$\frac{m}{s^{2}}$
*H*	Column height	m
*h*	Position	m
$\dot{h}$	Mean velocity	$\frac{m}{s}$ or $\frac{\mu m}{s}$
$v_{t}$	Velocity in the tube	$\frac{\mu m}{s}$
$v_{c}$	Velocity in the channel	$\frac{m}{s}$
$l_{c}$	Channel length	m
*w*	Channel width	m
*b*	Channel height, gap, or depth	μm
$l_{t}$	Tube length	m
*r*	Tube radius	m
$\eta_{0}$	Blood plasma viscosity	Pa·s
$\eta_{eff}$	Effective viscosity	
*m*	Consistency index	
*n*	Viscosity exponent	
K(m,n)	Generalized consistency index
$\eta_{htc}$	Normalized viscosity to hematocrit	
ϕ	Red blood cells concentration	
[η]	Intrinsic viscosity	
$\phi_{max}$	Maximum packing fraction	
$C_{0}$	Characteristic number	
$E_{0}$	Bending energy of a healthy RBC	J
*d*	Mean diameter of a RBC	μm
${\dot{\gamma }}_{0}$	Effective shear rate	s^−1^
κ	Variation coefficient	
$\sigma_{w}$	Wall stress	Pa
${\dot{\gamma }}_{w}$	Wall shear rate	s^−1^

## Appendix A. Comparison with Weissenberg–Rabinowitch–Mooney Correction

In this appendix, we present a comparison between our results for the effective viscosity and the Weissenberg–Rabinowitch–Mooney correction.

According to the Weissenberg–Rabinowitch–Mooney (WRM) correction, the wall stress in the tube is given as follows:

(A1) $$\sigma_{w}=\frac{r}{2l_{t}}\left(\rho gH-P_{cap}\right).$$

The wall shear rate associated with the non-Newtonian fluid is defined as:

(A2) $${\dot{\gamma }}_{w}={\dot{\gamma }}_{a}\frac{1}{4}\left(3+\frac{d\ln{\dot{\gamma }}_{a}}{d\ln\sigma_{w}}\right).$$

The apparent shear rate is defined as

(A3) $${\dot{\gamma }}_{a}=\frac{4Q}{\pi r^{3}},$$

where $Q=wb^{2}\left(\frac{\dot{h}}{b}\right)$.

For a power law model of non-Newtonian fluids, we can relate the wall stress with the apparent shear rate by means of

(A4) $$\sigma_{w}=m{\dot{\gamma }}_{a}^{n},$$

(A5) $$\frac{d\ln{\dot{\gamma }}_{a}}{d\ln\sigma_{w}}=\frac{1}{n}.$$

According to the WRM correction, the viscosity of the fluid will be given as

(A6) $$\eta =\frac{\sigma_{w}}{{\dot{\gamma }}_{w}}.$$

Combining Equations (A1), (A2), and (A6) we define an effective viscosity as the quotient between the viscosity of blood ($\eta_{b}$) and the viscosity of its plasma ($\eta_{p}$)

(A7) $$\begin{array}{ccc} \eta_{WRM} & = & \frac{\eta_{b}}{\eta_{p}}. \end{array}$$

For plasma n=1, therefore, the relation in Equation (A7) can be reduced to

(A8) $$\eta_{WRM}=\frac{4}{\left(3+\frac{1}{n}\right)}\frac{\Delta P_{eff_{b}}}{\Delta P_{eff_{p}}}\frac{{\dot{\gamma }}_{a_{p}}}{{\dot{\gamma }}_{a_{b}}}.$$

Substituting Equation (A3) in Equation (A8), we write the effective viscosity as

(A9) $$\eta_{WRM}=\frac{4}{\left(3+\frac{1}{n}\right)}\eta_{eff},$$

where the term $4/\left(3+\frac{1}{n}\right)$ is constant and does not affect the qualitative behavior of the viscosity.

To support this statement, we use Equation (12), and taking the logarithm from both sides of Equation (14), we see

(A10) $$\ln\eta_{eff}=\ln A\left(m,n\right)+\left(n-1\right)\ln\left(\frac{\dot{h}}{b}\right),$$

where we obtained that the slope of Equation (A10) determines the exponent (n−1), and A(m,n) corresponds to $K\left(m,n\right)/K_{p}\left(m_{p},n=1\right)$. Then, taking the logarithm from Equation (A9) and comparing with Equation (A10), they only differ in a constant parameter and do not affect the power law behavior.
